# Prognostic value of coronary flow reserve assessed by transthoracic Doppler echocardiography on long-term outcome in asymptomatic patients with type 2 diabetes without overt coronary artery disease

**DOI:** 10.1186/1475-2840-12-121

**Published:** 2013-08-27

**Authors:** Takayuki Kawata, Masao Daimon, Rei Hasegawa, Tomohiko Toyoda, Tai Sekine, Toshiharu Himi, Daigaku Uchida, Sakiko Miyazaki, Kuniaki Hirose, Ryoko Ichikawa, Masaki Maruyama, Hiromasa Suzuki, Hiroyuki Daida

**Affiliations:** 1 Department of Cardiology, Juntendo University School of Medicine, 2-1-1 Hongo, Bunkyo-ku, Tokyo 113-8421, Japan; 2 Department of Cardiovascular Science and Medicine, Chiba University Graduate School of Medicine, Chiba, Japan; 3 Kimitsu Chuo Hospital, Kisarazu, Japan

**Keywords:** Coronary flow reserve, Diabetes mellitus, Transthoracic doppler echocardiography

## Abstract

**Background:**

Cardiovascular risk stratification of asymptomatic diabetic patients is important and remains a difficult clinical problem. Our aim was to test the hypothesis that coronary flow reserve (CFR) assessed by noninvasive transthoracic Doppler echocardiography predicts prognosis in those patients.

**Methods:**

From February 2002 to January 2005, we evaluated 135 consecutive asymptomatic patients (74 male; mean age, 63 ± 9 years) with type 2 diabetes without a history of coronary artery disease. Adenosine triphosphate (0.14 mg/kg/min) stress Doppler echocardiography was performed to evaluate CFR of the left anterior descending artery. Patients with a CFR < 2.0 were also excluded based on the suspicion of significant coronary artery stenosis in the left anterior descending artery.

**Results:**

There were 111 patients (60 male; mean age, 64 ± 9 years) enrolled. During a median follow-up of 79 months, 20 events (5 deaths, 7 acute coronary syndromes, 8 coronary revascularizations) occurred. The optimal cut-off value of CFR to predict events was 2.5 (area under the receiver-operating characteristic curve = 0.65). Multivariate analysis showed that the independent prognostic indicators were male gender (p < 0.05) and a CFR < 2.5 (p < 0.01). Kaplan-Mayer analysis revealed that the event rate was significantly higher (log-lank, p < 0.01) in patients with CFR < 2.5 than in those with CFR ≥ 2.5.

**Conclusions:**

CFR obtained by transthoracic Doppler echocardiography provides independent prognostic information in asymptomatic patients with type 2 diabetes without overt coronary artery disease. Patients with CFR < 2.5 had a worse long-term outcome.

## Background

The prevention of cardiovascular complications is a crucial goal in the treatment of diabetes, because the incidence of coronary artery disease (CAD) in patients with type 2 diabetes is increasing worldwide as well as in Japan [1]. Likewise, it is important to identify patients at high risk for developing cardiovascular complications in order to reduce morbidity and mortality. However, cardiovascular risk stratification of patients with diabetes remains a difficult clinical problem.

Coronary flow reserve (CFR), estimated as the ratio of maximal hyperemic to basal coronary flow velocity, is an important physiological parameter in the coronary circulation that reflects the function of large epicardial arteries and the microcirculation. Previous reports have shown that noninvasive evaluation of CFR by transthoracic echocardiography is a useful tool to predict cardiovascular events in patients with cardiovascular diseases such as hypertension [2], CAD [3], and cardiomyopathy [4,5]. With regard to diabetes, Cortigiani and co-workers reported that CFR ≤ 2.0 in response to high-dose dipyridamole (0.84 mg/kg/min) provided prognostic information in diabetic and non-diabetic patients with known or suspected CAD [6]. Although they excluded patients with myocardial ischemia using dipyridamole stress echocardiography, they included patients with known CAD (i.e., history of myocardial infarction, coronary revascularization and/or angiographic evidence of > 50% diameter coronary stenosis) or regional wall motion abnormalities. They also reported in another paper [2] that CFR ≤ 1.91 was the best value for diagnosing coronary stenosis of ≥ 75% in hypertensive and normotensive patients (including diabetics) with known or suspected CAD. Therefore, in their study [6] of CFR in diabetic patients, patients with CFR ≤ 2.0 may have had significant coronary artery stenosis. As a result, the events that occurred during follow-up in their study included events due to diabetes per se and events due to CAD.

To avoid these limitations, we carefully enrolled asymptomatic patients with diabetes without a history of CAD. We tested the hypothesis that CFR assessed by noninvasive transthoracic Doppler echocardiography would predict cardiac events and prognosis, and would be useful to stratify cardiac risk in these selected patients.

## Methods

### Patients and protocol

The present study was a prospective, observational study. From February 2002 to January 2005, we enrolled 135 consecutive asymptomatic patients (74 male; mean age, 63 ± 9 years) with type 2 diabetes without a history of cardiovascular disease. Patients were included if they met the following inclusion criteria: outpatients with type 2 diabetes, no symptoms, no history of cardiovascular disease, and a clinically stable condition. All patients that met these inclusion criteria underwent two-dimensional echocardiography and a treadmill exercise test, and patients who had wall motion abnormalities, atrial fibrillation, left ventricular (LV) hypertrophy (wall thickness at end-diastole > 12 mm), valvular heart disease or a positive treadmill test were excluded from follow-up. Furthermore, patients with CFR < 2.0 were also excluded on the suspicion of significant coronary artery stenosis in the left anterior descending coronary artery (LAD) based on the results of previous studies [7,8].

All subjects were studied after an overnight fast and refrained from caffeine intake for more than 12 hours, since caffeine may modulate the effects of adenosine. Medical treatments, such as antihypertensive agents and statins were continued except antidiabetic agents during acquisition of all study data including treadmill testing. Venous blood sampling and coronary flow velocity measurements were carried out after informed consent was obtained. The outcome of the present study was clinical events during follow-up. Death from all causes, acute coronary syndrome (ACS) and coronary revascularization were considered clinical events. The protocol was approved by the committee on medical ethics and clinical investigation of Chiba University Hospital.

### Coronary risk factors

Venous blood samples were drawn from a peripheral vein immediately before coronary flow velocity measurements for determination of serum creatinine, fasting blood sugar, glycosylated hemoglobin, and lipid profiles (triglycerides, high-density lipoprotein cholesterol and low-density lipoprotein cholesterol). The diagnosis of diabetes was assured in all patients by determination of glucose in the fasting state based on the criteria of the World Health Organization [9]. Dyslipidemia was defined as low-density lipoprotein cholesterol ≥ 140 mg/dl or high-density lipoprotein cholesterol < 40 mg/dl or triglycerides ≥ 150 mg/dl or already receiving medical treatment. Arterial hypertension was defined as systolic blood pressure above 140 mmHg or diastolic blood pressure above 90 mmHg, or already receiving medical treatment. Smoking habit was confirmed by a personal interview.

### Treadmill exercise test

A treadmill exercise test was performed using the Bruce protocol. A 12-lead electrocardiogram was continuously monitored during the test, and blood pressure was recorded at rest and every 2 minutes during exercise and recovery. Exercise was terminated when subjects achieved their target heart rate, defined as 85% of the age-predicted maximal heart rate. Any change in the ST segment was automatically measured 80 ms after the J point with the Marquette system. The result was considered positive if the ST segment was horizontal or had a downslope with a 1-mm depression.

### Echocardiography

Echocardiographic examinations were performed with a SONOS 5500 digital ultrasound system (Philips Medical Systems, Andover, MA). LV ejection fraction was calculated with Simpson’s method using the 2 and 4 chamber views. M-mode echocardiography was performed to measure LV mass. The ultrasound beam was aligned perpendicular to the interventricular septum and the posterior wall at a level slightly below the mitral leaflet tips. LV mass was calculated from measurements of the septal (SD) and posterior wall thickness (PD), and LV dimensions (LVID) at end-diastole using the following formula [10]: 1.04 × ([SD + LVID + PD]^3^ − [LVID]^3^) – 13.6. The LV mass calculated using this formula was indexed to body surface area.

The method of CFR measurement with transthoracic Doppler echocardiography was described previously [7,8]. Coronary blood flow velocity in the distal portion of the LAD was measured with a high frequency transducer (5 to 7 MHz) using the guidance of color Doppler flow mapping. With a sample volume (2.5 or 3.0 mm wide) positioned on the color signal in the LAD, Doppler spectral tracings of flow velocity in the LAD were recorded using a fast Fourier transformation. We first recorded baseline spectral Doppler signals in the LAD, then intravenous adenosine was administered (0.14 mg/kg/min) to record spectral Doppler signals during hyperemia. Mean diastolic velocities (MDVs) were measured at baseline and peak hyperemic conditions from the Doppler signal recordings. Measurements were averaged over three cardiac cycles. CFR was defined as the ratio of hyperemic to basal MDV. To determine the reproducibility of MDV, a total of 10 randomly selected examinations were analyzed twice by one investigator at a 1-week interval and once by another investigator.

### Follow-up data

Outcome was obtained from hospital chart reviews, and telephone interviews with the referring physician. The clinical events recorded during follow-up were all-cause death, nonfatal ACS and clinically indicated coronary revascularization (surgery or angioplasty). If more than one of these events occurred in the same patient, the patient was censored at the time of the most severe event.

### Statistical analysis

Baseline characteristics were expressed as the mean ± standard deviation or number (%). Prediction of events was evaluated by receiver operating characteristic curve analysis. The cumulative event-free rates were obtained from Kaplan-Meier estimates, and differences were tested by the log-rank test. The association of selected variables with outcome was assessed with a univariate Cox proportional hazards model, and the variables that were significant in the univariate model were then entered into a multivariate Cox model using a forward stepwise method. Hazard ratios (HR) with the corresponding 95% confidence intervals (CI) were estimated. Comparison of baseline characteristics between the groups stratified according to CFR was performed with an unpaired *t* test; a chi-square test was used for categorical variables. A probability value < 0.05 was considered significant. All data were statistically analyzed using JMP version 8.0 (SAS Institute, Cary, NC, USA).

## Results

All patients who had a negative treadmill exercise test achieved their target heart rate. There were no adverse reactions to adenosine such as atrioventricular block, chest pain, flushing or palpitations during coronary flow measurement. Five patients were excluded based on their treadmill exercise test, and 2 more were excluded due to wall motion abnormalities. We could not obtain LAD blood flow in 9 patients, and 7 had CFR < 2.0. Furthermore, one patient refused to participate in the study. Therefore, we enrolled 111 patients (60 male; mean age, 64 ± 9 years; BMI, 24.2 ± 3.6 kg/m^2^) that had adequate coronary flow recordings for the assessment of CFR. The interobserver and intraobserver variabilities for the measurement of Doppler velocity were 5.0% and 3.9%, respectively.

During a median follow-up of 79 months (range, 2 to 112 months), 20 events (5 deaths, 7 ACSs, 8 coronary revascularizations) occurred (event rate, 3.3%/year). From a receiver-operating characteristics curve analysis, the optimal cut-off value of CFR to predict events was 2.5 (area under the receiver-operating characteristic curve = 0.65). Table 1 presents patients’ general characteristics, the results of venous blood tests, and hemodynamic and echocardiographic data in patients stratified according to CFR (≥ 2.5 and < 2.5). Compared with patients with CFR ≥ 2.5, the general characteristics of the patients with CFR < 2.5 were similar except for age, hyperemic MDV and CFR. Table 2 presents the occurrence rate of events in relation to CFR values.

**Table 1:** Patients’ general characteristics and echocardiographic data

**Variables**	**CFR ≥ 2.5, n = 82**	**CFR < 2.5, n = 29**	**p**
Age (years)	62 ± 9	67 ± 9	0.02
Male	43 (52.0)	17 (59.0)	0.57
BMI (kg/m^2^)	24.5 ± 3.7	23.3 ± 3.1	0.11
FBS (mmol/l)	8.6 ± 2.6	8.4 ± 2.2	0.79
HbA1c (%) [NGSP]	7.7 ± 1.2	7.7 ± 1.0	0.90
eGFR (ml/min./1.73 m^2^)	80.6 ± 16.9	77.2 ± 19.3	0.39
Hypertension	48 (59.3)	19 (63.3)	0.51
Dyslipidemia	43 (53.1)	15 (50.0)	0.95
Smoking	15 (18.5)	6 (20.0)	0.78
Diabetes treatment			
Sulfonylurea	45 (55.6)	15 (50.0)	0.77
Biguanide	16 (19.8)	8 (26.7)	0.36
Thiazolidinedione	24 (29.6)	5 (16.7)	0.21
alpha-glucosidase inhibitor	36 (44.4)	7 (23.3)	0.06
Insulin	13 (16.0)	6 (20.0)	0.55
Other treatment			
Statin	21 (25.9)	9 (30.0)	0.57
antihypertensive agents	38 (46.3)	13 (44.8)	0.89
EF (%)	67.7 ± 6.2	66.3 ± 6.1	0.56
LVMI (g/m^2^)	98.9 ± 23.3	88.9 ± 27.2	0.32
MDV baseline (cm/sec)	20 ± 5	22 ± 5	0.08
MDV hyperemia (cm/sec)	62 ± 14	52 ± 13	0.002
CFR	3.09 ± 0.38	2.33 ± 0.13	<0.001

Data are presented as number (%) or mean ± SD.

Abbreviations: *BMI* body mass index, *FBS* fasting blood sugar,*HbA1c* glycated hemoglobin, *eGFR* estimated glomerular filtration rate, *EF* ejection fraction, *LVMI* left ventricular mass index, *SD* standard deviation.

**Table 2:** Event rates in patients stratified by CFR

	**CFR ≥ 2.5, n = 82**	**CFR < 2.5, n = 29**
All cause death	2 (2.4)	3 (10.3)
Acute coronary syndrome	3 (3.6)	4 (13.8)
Revascularization	3 (3.6)	5 (17.2)

Data are presented as number (%).

**Figure 1:**
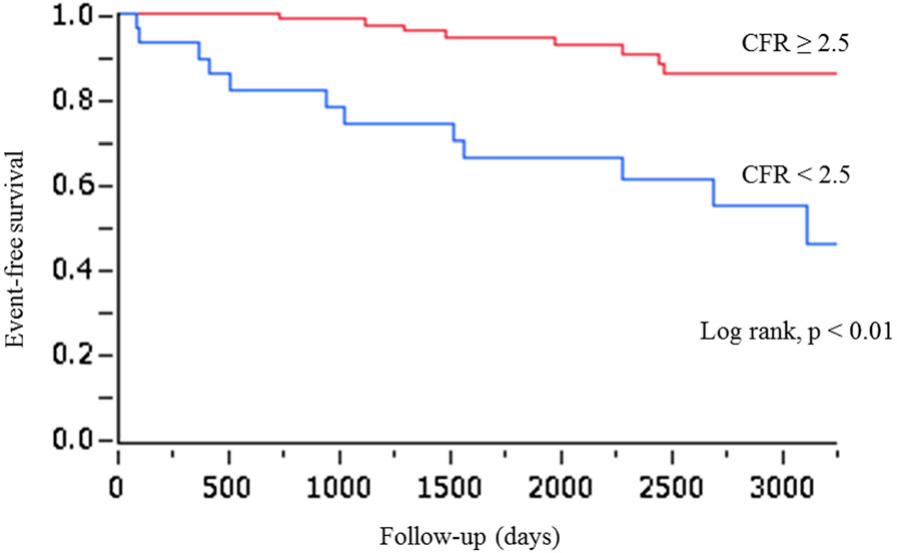
Kaplan-Meier curves for all events (all cause death, ACS and revascularization) in patients stratified according to their CFR (≥ 2.5 or < 2.5).

Kaplan-Meier analysis revealed that the event rate was significantly higher (log-lank, p < 0.01) in patients with CFR < 2.5 than in patients with CFR ≥ 2.5 (Figure 1). Univariate and multivariate predictors of events are summarized in Table 3. Univariate analysis showed that age (HR = 1.06, 95% CI = 1.01-1.12, p < 0.05), male gender (HR = 2.59, 95% CI = 1.04-7.35, p < 0.05) and CFR < 2.5 (HR = 4.89, 95% CI = 2.02-12.5, p < 0.001) were predictors of outcome. In multivariate analysis, CFR < 2.5 was the strongest independent predictor of future events (HR = 3.95, 95% CI = 1.60-10.3, p < 0.01), followed by male gender (HR = 2.57, 95% CI = 1.03-7.29, p < 0.05).

**Table 3:** Univariate and multivariate predictors of outcome

	**Univariate**		**Multivariate**	
	HR (95% CI)	p	HR (95% CI)	p
Age	1.06 (1.01-1.12)	0.025	1.04 (0.99-1.10)	0.11
Male gender	2.59 (1.04-7.35)	0.042	2.57 (1.03-7.29)	0.044
Hypertension	1.51 (0.61-4.27)	0.39		
Dyslipidemia	1.51 (0.61-4.03)	0.37		
Smoking	1.19 (0.38-3.14)	0.75		
HbA1c	0.97 (0.63-1.42)	0.88		
eGFR	1.00 (0.97-1.03)	0.96		
EF	0.88 (0.68-1.09)	0.27		
LVMI	1.03 (0.98-1.09)	0.25		
CFR < 2.5	4.89 (2.02-12.5)	<0.001	3.95 (1.60-10.3)	0.003

abbreviations as in Table 1.

**Figure 2:**
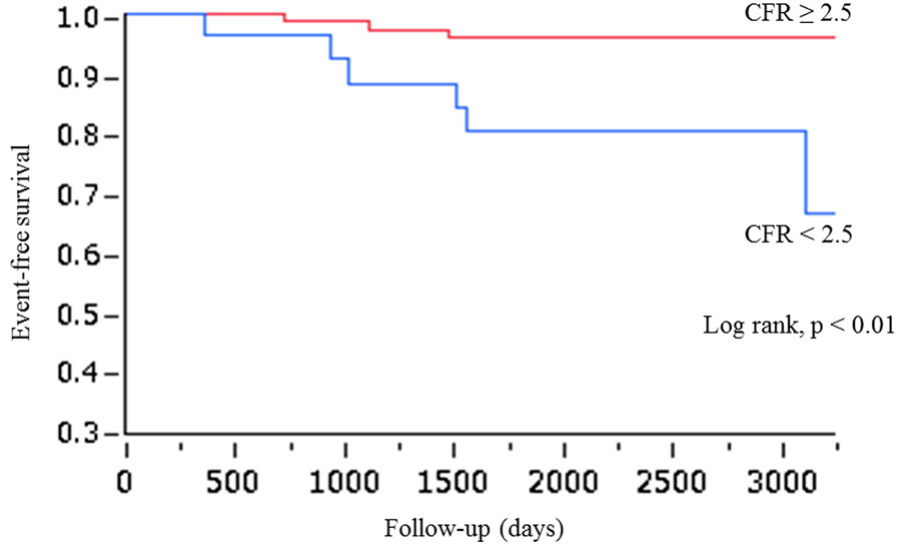
Kaplan-Meier curves for cardiac hard events (cardiac death and ACS) in patients stratified according to their CFR (≥ 2.5 or < 2.5).

Cardiac death and ACS (cardiac hard events) occurred in 9 cases (event rate, 1.4%/year). A univariate Cox model (Table 4) showed that the predictors of cardiac hard events were CFR < 2.5 (HR = 4.99, 95% CI = 1.22-24.4, p < 0.05), age (HR = 1.09, 95% CI = 1.01-1.19, p < 0.05) and estimated glomerular filtration rate (HR = 0.96, 95% CI = 0.92-0.99, p < 0.05). Although multivariate analysis failed to show CFR < 2.5 as an independent predictor of cardiac hard events (Table 4), a chi-square test (Fisher’s exact probability test) indicated that CFR < 2.5 was significantly related to cardiac hard events (p < 0.01). In addition, Kaplan-Meier analysis showed that the event rate was significantly higher (log-lank test, p < 0.01) in patients with CFR < 2.5 than in patients with CFR ≥ 2.5 (Figure 2).

**Table 4:** Univariate and multivariate predictors of cardiac hard events

	**Univariate**		**Multivariate**	
	HR (95% CI)	p	HR (95% CI)	p
Age	1.09 (1.01-1.19)	0.035	1.05 (0.96-1.15)	0.29
Male	3.01 (0.69-20.6)	0.15		
Hypertension	1.88 (0.43-12.8)	0.42		
Dyslipidemia	0.73 (0.17-3.12)	0.66		
Smoking	0.55 (0.03-3.15)	0.55		
HbA1c	0.92 (0.42-1.66)	0.80		
eGFR	0.96 (0.92-0.99)	0.035	0.98 (0.94-1.02)	0.34
EF	0.65 (0.28-1.06)	0.10		
LVMI	1.03 (0.97-1.13)	0.33		
CFR < 2.5	4.99 (1.22-24.4)	0.026	3.47 (0.83-17.4)	0.089

abbreviations as in Table 1.

## Discussion

The key finding of the present study was that in asymptomatic patients with type 2 diabetes without overt CAD, CFR assessed by transthoracic Doppler echocardiography provides long-term prognostic information noninvasively. Compared with CFR ≥ 2.5, patients with CFR < 2.5 had significantly worse outcome. Moreover, CFR < 2.5 was a strong independent predictor of outcome.

### Diabetes and coronary artery disease

The prevalence of diabetes in the Japanese population is rapidly increasing, and the incidence of cardiac disease in diabetes is also rising. In fact, the event rate in this study was higher than that reported in previous studies [1,11]. Because CAD is a major health concern and the leading cause of death in patients with type 2 diabetes, the prevention of CAD is an urgent task of clinicians.

Intensive glycemic control is the basis of diabetes management to prevent complications such as micro- and macro-vascular disease. However, the treatment of diabetes is sometimes difficult because some large clinical trials showed that intensive glucose lowering treatment does not always reduce coronary events [12-14]. Although the current standard of care for diabetes emphasizes the reduction of multiple coronary risk factors, there has also been substantial interest in the identification of asymptomatic patients at high risk for developing CAD in order to optimize therapeutic interventions. Using CFR, we tried to identify the high risk patients with diabetes. As indicated in Table 1, the general characteristics including diabetes control of patients with CFR ≥ 2.5 and CFR < 2.5 were similar. However, Figure 1 showed that the event rate was markedly higher in patients with CFR < 2.5 than in patients with CFR ≥ 2.5, indicating that CFR is a valuable tool to identify high-risk diabetic patients without overt CAD.

### CFR and coronary events

Based on the report [15] by Gould et al., CFR has been widely used as an important index of epicardial coronary artery stenosis. However, CFR also reflects the function of the coronary microcirculation in patients without epicardial coronary artery stenosis [16]. Reduced CFR has been demonstrated in diabetic patients without overt CAD because of functional and structural alterations of the coronary microcirculation [17,18]. Reduced CFR has also been shown even in patients with prediabetes, those with impaired fasting glucose and those with glucose intolerance [19]. These abnormalities may lead to impaired vasodilator function, and this may contribute to adverse future cardiovascular events. In fact, many previous reports have shown that impaired vasodilator function assessed by invasive methods adversely affects prognosis [20-22]. Therefore, assessment of CFR is considered a reasonable method to stratify the risk of future coronary events in diabetic patients.

### Comparison between adenosine and dipyridamole, as well as CFR cut-off value

Both adenosine and dipyridamole have been widely used to induce hyperemic coronary flow and to measure CFR. Adenosine dilates coronary arterioles through adenosine A2 receptors by increasing adenylate cyclase and decreasing calcium uptake [23]. Dipyridamole inhibits the cellular uptake of adenosine and indirectly dilates coronary arteries [24]. Kozàkovà et al. [25] compared the CFR values obtained with adenosine (0.14 mg/kg/min) and dipyridamole at high (0.84 mg/kg/min) and low (0.56 mg/kg/min) doses and found that the CFR measured with adenosine was equivalent to the CFR measured with high-dose dipyridamole. Lim et al. [26] also found the same results as Kozàkovà et al. Therefore, the CFRs measured with adenosine and high-dose dipyridamole are equivalent.

In the present study, patients with CFR < 2.0, a cut-off value used in previous studies that investigated the prognostic value of reduced CFR on outcomes, were excluded. Previous studies have reported that CFR < 2.0 with adenosine reflects not only significant epicardial coronary artery stenosis [7], but also physiological myocardial ischemia [8]. Although microcirculatory dysfunction in patients with diabetes causes reduced CFR, it has also been reported that CFR < 2.0 obtained by transthoracic Doppler echocardiography had a high predictive value for the presence of significant LAD stenosis, even in a population that included patients with diabetes [27]. On the other hand, the CFR in an infarct-related vessel is generally low [28,29], because of microvascular injury and a reduction in the size of the coronary vascular bed. Therefore, although a CFR < 2.0 is not a precise diagnostic marker for significant coronary artery stenosis or CAD, we thought there was a strong likelihood that patients with CFR < 2.0 would have significant coronary artery stenosis or CAD. For that reason, it may be expected that CFR < 2.0 measured with high-dose dipyridamole would reflect a poor prognosis when the study population includes patients with known CAD and wall motion abnormalities [6]. Because we wanted to stratify the high-risk patients with diabetes but without symptoms or overt CAD in the present study, it is appropriate that patients with CFR < 2.0 were excluded from the study.

### CFR assessed by transthoracic Doppler echocardiography

Recent developments in transthoracic Doppler echocardiography technology enable the noninvasive visualization of coronary blood flow and the assessment of CFR. Using a high-frequency transducer and a reduced velocity range in color Doppler echocardiography, it is possible to obtain a high success rate of CFR measurement [7,8]. This technique has been validated by an intracoronary Doppler flow wire technique advanced into the coronary vessels [30]. Because transthoracic Doppler echocardiography is noninvasive and does not use radiation, it is useful and suitable to evaluate the coronary microcirculation in patients without an indication for coronary angiography [31,32]. Moreover, echocardiography is much simpler to perform and less expensive than myocardial scintigraphy. This technique is now used worldwide and is established as a noninvasive tool to assess the coronary circulation [33-35].

### Clinical implications

A previous report has shown that half of patients with asymptomatic type 2 diabetes with microalbuminuria but without cardiac disease had significant atherosclerosis [36]. Furthermore, patients with type 2 diabetes without previous myocardial infarction had as high a risk of myocardial infarction as nondiabetic patients with previous myocardial infarction [37]. To select appropriate management strategies, risk stratification for future events is essential in diabetic patients. Screening by noninvasive CFR is a valuable, safe and inexpensive tool. On the basis of our results, patients with CFR < 2.5 have an elevated risk of future events. Therefore, risk factor control in high-risk asymptomatic patients with type 2 diabetes is important, with tight glucose regulation and the use of renin-angiotensin system blockers, aspirin and lipid-lowering agents [38]. Moreover, the CFR value might change during follow-up, resulting from these interventions or disease progression, and the periodic use of this noninvasive assessment in clinical practice might help to manage asymptomatic type 2 diabetes.

### Limitations

There are some limitations of the present study. First, CFR was measured only in the LAD. Although measurement of CFR in three coronary arteries is preferable, it remains technically challenging at the present time. However, coronary microcirculatory dysfunction affects the left ventricle globally as well as regionally [39], and therefore CFR assessment in the LAD is an excellent option for evaluating the global coronary microcirculation. Second, we excluded the presence of CAD among the study subjects only by a standard exercise tolerance test and CFR < 2.0 with Doppler echocardiography. Compared with wall motion assessment by stress echocardiography or perfusion assessment by stress myocardial scintigraphy, a standard exercise tolerance test has low sensitivity and specificity to detect CAD. However, all of the studied patients had both a negative exercise test and CFR ≥ 2.0, indicating that there was no significant LAD stenosis [7,8,27]. Third, although we did not focus on diastolic function, diastolic dysfunction leads to impairment of CFR. Various degrees of diastolic dysfunction have been reported to occur in most patients with diabetes [40], and diastolic dysfunction might have affected CFR in the present study. Moreover, hypertension and prehypertension is related to the reduction of CFR. However, no significant difference was observed in CFR between patients with and without hypertension in the present study (2.88 ± 0.50 vs. 2.90 ± 0.44, p = 0.85). This result may be because of the small number of patients in the study and/or the effect of treatment on hypertension. Further investigation of patients with diabetes but without hypertension or prehypertension would provide new insight into this issue. Lastly, the number of the study subjects was small compared with previous study [6]. As a matter of fact, multivariate analysis failed to show CFR < 2.5 as an independent predictor of cardiac hard events, probably because of the small number of subjects included in this study. We carefully selected asymptomatic diabetic patients that conformed to our purpose although the subject number was small. However, large-scale studies are necessary to confirm our findings.

## Conclusion

CFR obtained by transthoracic Doppler echocardiography provides independent prognostic information in asymptomatic patients with type 2 diabetes without overt CAD. Because patients with CFR < 2.5 had a worse outcome, a more aggressive intervention is warranted in these patients with better control of coronary risk factors and more frequent follow-up by noninvasive stress testing.

## Competing interest

No potential conflicts of interest relevant to this article were reported.

## Authors’ contributions

TK and MD designed the study, participated in data collection and wrote the manuscript. RH, TT, TS, TH and DU participated in data collection and discussed and reviewed the manuscript. SM, KH, RI, MM, HS and HD reviewed the manuscript. All authors read and approved the final manuscript.
